# Supplementary material for: Use of Leaves as Bioindicator to Assess Air Pollution Based on Composite Proxy Measure (APTI), Dust Amount and Elemental Concentration of Metals
Source: Plants (Basel). 2020 Dec 9;9(12):1743. doi: 10.3390/plants9121743 (PMC7764743; doi:10.3390/plants9121743)
Supplement: Supplementary file 1 [file plants-09-01743-s001.pdf]

Use of Leaves as Bioindicator to Assess Air Pollution Based on Composite Proxy Measure (APTI). Dust Amount and Elemental Concentration of Metals

Vanda Éva Molnár [1]. Dávid Tőzsér [2]. Szilárd Szabó [1]. Béla Tóthmérész [3] and Edina Simon [2,*]

[1] Department of Physical Geography and Geoinformatics. University of Debrecen. Debrecen. H−4032 Hungary; molnarvandaeva@gmail.com (V.É.M.); szabo.szilard@science.unideb.hu (S.S.)

[2] Department of Ecology. University of Debrecen. Debrecen. H−4032. Hungary; tozser.david@windowslive.com

[3] MTA−DE Biodiversity and Ecosystem Services Research Group. Debrecen. H−4032. Hungary; tothmerb@gmail.com

* Correspondence: edina.simon@gmail.com

Table S1. Dust concentration and APTI values of the study sites.

	T.europaea						*C.occidentalis*					
	June			September			June			September		
	urban	industrial	rural	urban	industrial	rural	urban	industrial	rural	urban	industrial	rural
coarse dust. μg cm^{-2}	36 ± 11	41 ± 14	17 ± 4	58 ± 29	48 ± 9	64 ± 8	24 ± 3.4	18 ± 7	11 ± 2	40 ± 13	70 ± 34	76 ± 3
fine dust. μg cm^{-2}	5.1 ± 0.6	4.2 ± 0.5	2.1 ± 0.2	4.9 ± 2.5	2.0 ± 0.4	1.2 ± 0.1	3.8 ± 0.3	1.6 ± 0.1	0.9 ± 0.1	5.5 ± 0.3	3.0 ± 0.4	4.9 ± 0.5
ascorbic acid. mg g^{-1}	0.9 ± 0.1	1.0 ± 0.1	0.7 ± 0.1	1.6 ± 0.3	1.2 ± 0.1	1.3 ± 0.2	1.8 ± 0.3	2.6 ± 0.4	2.2 ± 0.1	3.0 ± 1.0	2.3 ± 0.9	2.8 ± 0.4
total chlorophyll. mg g^{-1}	6.5 ± 0.4	7.4 ± 0.7	8.3 ± 1.1	5.8 ± 1.4	7.0 ± 0.1	7.9 ± 1.9	10 ± 1	7.4 ± 0.5	17 ± 1	5.4 ± 1.2	3.9 ± 1.9	8.1 ± 0.4
pH	6.5 ± 0.1	6.6 ± 0.1	6.5 ± 0.1	7.1 ± 0.1	6.7 ± 0.1	6.5 ± 0.1	8.9 ± 0.2	9.0 ± 0.2	8.9 ± 0.2	9.3 ± 0.2	6.3 ± 3.1	9.3 ± 0.1
relative water content. %	72 ± 1	78 ± 4	60 ± 4	78 ± 3	76 ± 2	67 ± 4	73 ± 1	76 ± 3	72 ± 5	93 ± 1	61 ± 30	97 ± 1
APTI	8.3 ± 0.1	9.2 ± 0.5	7.1 ± 0.5	10 ± 1	9.2 ± 0.1	8.6 ± 0.3	11 ± 1	12 ± 1	13 ± 1	14 ± 1	9.5 ± 4.4	15 ± 1

Table S2. Elemental concentration in leave's tissue at the study sites.

	T. europaea			*C. occidentalis*			
	urban	industrial	rural	urban	industrial	rural	LoD. ug g^{-1}
Al. mg kg^{-1}	129 ± 17	565 ± 19	181 ± 46	112 ± 9	157 ± 11	133 ± 23	300
Ba. mg kg^{-1}	4.5 ± 0.5	10.7 ± 0.3	16.6 ± 3.3	11.3 ± 2.4	20.1 ± 1.5	28.2 ± 2.4	2.5
Ca. g kg^{-1}	14.6 ± 0.8	15.1 ± 1.1	13.2 ± 1.9	44.7 ± 2.9	37.1 ± 5.6	23.7 ± 2.5	5
Cd. mg kg^{-1}	0.08 ± 0.08	0.08 ± 0.08	0.12 ± 0.001	n.d.	n.d.	n.d.	25
Co. mg kg^{-1}	0.04 ± 0.04	0.04 ± 0.04	0.12 ± 0.001	0.12 ± 0.002	0.04 ± 0.04	0.04 ± 0.04	150
Cr. mg kg^{-1}	0.83 ± 0.15	2.93 ± 0.25	0.7 ± 0.21	0.66 ± 0.05	0.87 ± 0.06	0.55 ± 0.05	50
Cu. mg kg^{-1}	4.2 ± 0.5	5.7 ± 0.5	6.9 ± 0.5	6.5 ± 0.8	7.3 ± 0.9	9.6 ± 0.8	50
Fe. mg kg^{-1}	181 ± 10	581 ± 11	215 ± 38	201 ± 11	231 ± 23	179 ± 22	50
K. g kg^{-1}	17.6 ± 2.0	10.0 ± 1.6	14.1 ± 1.3	15.9 ± 3.7	11.6 ± 1.3	12.6 ± 1.1	50
Mg. g kg^{-1}	5.0 ± 1.0	4.8 ± 0.3	3.2 ± 0.4	4.0 ± 0.4	4.9 ± 0.6	2.7 ± 0.2	2.5
Mn. mg kg^{-1}	31.4 ± 10.4	52.9 ± 5.4	99.7 ± 18	49.6 ± 7.6	65.0 ± 7.8	79.5 ± 6.4	2.5
Na. mg kg^{-1}	66.5 ± 0.6	132 ± 6	131 ± 16	84.5 ± 13.5	137 ± 7.8	177 ± 17	5
Ni. mg kg^{-1}	1.32 ± 0.44	1.3 ± 0.04	1.07 ± 0.08	0.82 ± 0.08	0.75 ± 0.12	0.76 ± 0.05	25
Pb. mg kg^{-1}	0.33 ± 0.11	0.69 ± 0.11	0.41 ± 0.07	0.37 ± 0.09	0.34 ± 0.09	0.34 ± 0.04	225
Sr. mg kg^{-1}	31.8 ± 5.4	68.9 ± 3.2	39.7 ± 6.9	79.9 ± 7.3	151.2 ± 33.1	42.1 ± 2.3	550
Zn. mg kg^{-1}	9.7 ± 0.7	27.2 ± 12.6	13.2 ± 1.5	14.0 ± 1.4	12.1 ± 0.8	13.0 ± 2.6	0.5

Notations: n.d. means concentration was below detection limit.

Table S3. Correlation (r_s) between elemental concentration, dust, and APTI values.

	T. europaea			*C. occidentalis*		
	coarse dust	fine dust	APTI	coarse dust	fine dust	APTI
coarse dust	1.000	0.633	0.661	1.000	**0.750**	0.000
fine dust	0.633	1.000	**0.828**	**0.750**	1.000	−0.583
Al	0.467	0.217	0.502	0.238	0.024	0.357
Ba	−0.650	**−0.800**	−0.594	−0.595	**−0.905**	**0.810**
Ca	−0.100	0.083	0.042	**0.810**	**0.952**	**−0.714**
Cd	−0.226	−0.261	0.044	n.d.	n.d.	n.d.
Co	−0.261	−0.183	−0.494	−0.051	0.355	−0.482
Cr	0.617	0.467	**0.803**	**0.714**	0.429	0.024
Cu	−0.583	−0.567	−0.410	−0.643	**−0.810**	0.571
Fe	0.367	0.267	0.494	0.429	0.238	0.071
K	−0.150	0.200	−0.117	−0.452	−0.190	−0.048
Mg	0.367	0.450	0.586	**0.762**	0.548	−0.143
Mn	**−0.733**	**−0.800**	**−0.669**	−0.429	**−0.738**	0.667
Na	−0.117	−0.117	0.109	−0.667	**−0.929**	**0.833**
Ni	0.333	0.200	0.644	0.571	0.429	−0.095
Pb	0.317	0.500	0.536	0.214	0.000	0.381
Sr	0.133	−0.017	0.335	**0.833**	0.548	−0.095
Zn	−0.050	−0.150	0.059	−0.071	0.143	−0.429

Bold letters indicate significant correlation values. n.d. means elemental concentration was not detected.
